# Infection control practices in Indian dental clinics: A cross-sectional study

**DOI:** 10.6026/973206300222049

**Published:** 2026-04-30

**Authors:** Braj Bhushan Mall, Waikhom Robindro Singh, Shamurailatpam Priyadarsini, Thingujam Debica, Bankim Ningthoujam, Haripriya Nongthombam, DP Ramizan

**Affiliations:** 1 Department of Oral & Maxillofacial Surgery, Dental College, Regional Institute of Medical Sciences (RIMS), Imphal 795004, Manipur, India; 2 Department of Conservative Dentistry and Endodontics, Dental College, RIMS, Imphal, Manipur, India; 3 Department of Prosthodontics and Crown & Bridge, Dental College, RIMS, Imphal, Manipur, India; 4 Department of Oral & Maxillofacial Surgery, Dental College, RIMS, Imphal, Manipur, India; 5 Department of Orthodontics, Dental College, RIMS, Imphal, Manipur, India; 6 Consulting Physician, Private Practice, Imphal, Manipur, India

**Keywords:** Infection control, dental clinics, disease transmission

## Abstract

Inadequate infection control in dental clinics increases occupational and patient risk of blood-borne and aerosol-transmitted infections. Therefore, it is of interest to evaluate infection control awareness and practices among 70 private dental clinics in India using a standardized questionnaire based on CDC guidelines. Overall adherence was moderate, with significant gaps in aerosol management, post- exposure protocols, sterilization practices and vaccination awareness. MDS-qualified practitioners demonstrated significantly higher compliance than BDS practitioners across multiple domains (p<0.05). Thus, we show the need for structured continuing education, stricter protocol enforcement and standardized infection control monitoring in private dental settings.

## Background:

Infection control in dental practice is critical due to routine exposure to blood, saliva and aerosolized microorganisms during clinical procedures [1]. Recent evidence confirms that dental aerosols can transmit respiratory and blood- borne pathogens, including HBV, HCV and emerging viral infections, particularly in inadequately ventilated environments [2]. The COVID-19 pandemic further highlighted structural deficiencies in infection prevention systems within outpatient healthcare settings, including dentistry [3]. Despite established international guidelines from the CDC and WHO, adherence to standard precautions remains inconsistent across private dental practices, especially in low-resource and peripheral regions [4]. Studies report persistent deficiencies in hand hygiene compliance, sterilization validation, vaccination awareness and biomedical waste segregation among dental professionals [5]. Inadequate knowledge regarding post-exposure prophylaxis and improper sharps disposal continue to pose occupational hazards [6]. Professional qualification and postgraduate training have been shown to influence infection control compliance, with specialists demonstrating higher adherence to protocol-driven practice [7]. Therefore, it is of interest to evaluate infection control awareness and practices among private dental practitioners in Manipur and to compare compliance patterns between BDS- and MDS-qualified clinicians.

## Materials and Methods:

This cross-sectional descriptive study was conducted among private dental clinics in the Imphal Valley, Manipur, following institutional ethical approval (Ref No. A/206/REB/PMOP (SP) 78/54/2019). Seventy private dental clinics were selected using a snowball sampling technique. Clinic owners holding either Bachelor of Dental Surgery (BDS) or Master of Dental Surgery (MDS) qualifications were included. Data were collected using a structured, self-administered questionnaire developed in accordance with CDC infection prevention guidelines and recent peer-reviewed literature. The questionnaire consisted of four demographic variables and 36 items assessing infection control practices. Domains included hand hygiene, personal protective equipment, routes of disease transmission, aerosol management, dental unit waterline biofilm control, sterilization practices, disinfection methods, vaccination awareness, post-exposure protocols, sharps disposal and environmental surface decontamination. Responses were categorized as correct or incorrect based on CDC standards and contemporary evidence- based guidelines. Overall performance scores were calculated as percentages and classified as Good (>70%), Average (51-70%), or Poor (≤50%). Data were entered using EpiData 3.1 and analyzed using SPSS software. Descriptive statistics were used to summarize demographic variables and response distributions. Pearson's Chi-square test was applied to compare BDS and MDS practitioners. A p-value <0.05 was considered statistically significant.

## Results:

Seventy private dental practitioners participated in the study, of whom 75.7% were BDS-qualified and 24.3% were MDS-qualified. The majority of respondents (61.4%) were aged between 25-35 years and 52.9% had long-standing clinical experience. Overall infection control performance was categorized as average, with notable variability across domains. Knowledge regarding glove use was high (84.3%), yet compliance with correct hand hygiene practices was inconsistent despite universal agreement on preference for liquid soap. Awareness of transmission routes was suboptimal, particularly regarding patient-to-patient transmission and disease spread through hand lesions. Knowledge related to aerosol-associated diseases was limited, with only 17.1% correctly identifying diseases transmitted via aerosols. Although 94.3% recognized that aerosols contain microorganisms, practical mitigation strategies such as rubber dam use (12.9%) and pre- procedural mouth rinses (1.4%) were poorly adopted. Biofilm awareness was low, with only 14.3% correctly identifying dental unit waterline biofilm formation sites, although 94.3% were aware of chemical or flushing-based control methods. Vaccination knowledge demonstrated mixed results; while 98.6% correctly identified the hepatitis B vaccination schedule, only 31.4% recognized that no vaccine exists for hepatitis C. None of the participants demonstrated comprehensive knowledge of post-exposure protocols following needlestick injuries. Sterilization practices showed significant gaps, as only 44.3% reported autoclaving airotor handpieces between patients and only 37.1% followed accepted sterilization protocols. Disposal of sharps revealed unsafe practices, with 50% demonstrating incorrect disposal responses and 32.9% showing improper needle recapping knowledge. Environmental surface disinfection compliance was comparatively high, with 92.9% reporting regular chair disinfection. Across multiple parameters, MDS practitioners demonstrated statistically significantly better compliance than BDS practitioners (p<0.05), particularly in transmission knowledge, biofilm awareness, sharps management and sterilization practices. Table 1 (see PDF) shows that the majority of participants were aged 25-35 years (61.4%), predominantly BDS-qualified (75.7%), with 52.9% reporting long-standing clinical experience. Table 2 (see PDF) demonstrates high awareness of glove use (84.3%) but comparatively lower correct identification of appropriate hand hygiene practices (75.7%) and statistically significant differences in splash protection practices (p<0.05). Table 3 (see PDF) indicates poor overall knowledge of transmission pathways, particularly patient-to-patient transmission (30.0%) and disease spread via hand lesions (22.9%), with significant qualification-based differences in selected domains. Table 4 (see PDF) shows very low recognition of biofilm formation sites (14.3%) despite high awareness of control measures (94.3%). Table 5 (see PDF) demonstrates limited awareness of aerosol-related diseases (17.1%) despite widespread recognition that aerosols contain microorganisms (94.3%). Table 6 (see PDF) indicates excellent awareness of hepatitis B vaccination schedules (98.6%) but complete absence of comprehensive post-exposure protocol knowledge (0%). Table 7 (see PDF) shows inadequate sharps management practices, with only 32.9% correctly avoiding needle recapping. Table 8 (see PDF) demonstrates suboptimal operational sterilization compliance, with only 44.3% reporting autoclave sterilization of airotors, less than one-third regularly validating autoclave effectiveness or servicing equipment appropriately and significant qualification-based differences observed across multiple sterilization parameters. Table 9 (see PDF) indicates high compliance with environmental surface disinfection (92.9%) but poor knowledge of blood spill management (15.7%). Table 10 (see PDF) shows moderate awareness of disinfectant effectiveness, with 74.3% correctly identifying chemicals active against viruses and spores.

## Discussion:

This cross-sectional assessment reveals moderate overall compliance with infection control practices among private dental practitioners in Manipur, with significant deficiencies in critical operational domains [8]. Although awareness of glove use and hepatitis B vaccination was high, substantial gaps were identified in aerosol management, sterilization validation, sharps handling and post-exposure protocols [9]. These findings indicate that foundational infection control knowledge does not consistently translate into comprehensive procedural adherence [10]. Aerosol-related awareness was particularly inadequate, with only a small proportion of practitioners correctly identifying aerosol- transmitted diseases despite widespread recognition that aerosols contain microorganisms [11]. Limited use of rubber dam isolation and pre-procedural mouth rinses further reflects insufficient mitigation of aerosol contamination during routine procedures [11]. Given the documented risk of airborne transmission in dental settings, these deficiencies may increase both occupational and cross-infection risk [12]. Sterilization practices demonstrated concerning operational weaknesses. Less than half of practitioners reported autoclaving airotor handpieces between patients and less than one-third routinely validated autoclave performance or adhered to recommended servicing intervals [13]. These findings suggest lapses not merely in awareness but in quality assurance and equipment monitoring systems, which are essential for preventing instrument-mediated transmission of infection [14]. The low awareness of contemporary sterilization methods further emphasizes the need for continuing professional education [15]. Sharps management practices were also suboptimal, particularly regarding needle recapping, a preventable cause of needlestick injury [16]. The absence of comprehensive knowledge regarding post-exposure prophylaxis protocols is clinically significant, as it may delay timely intervention following occupational exposure [17].

In contrast, environmental surface disinfection compliance was comparatively higher, suggesting that visible cleaning practices are more consistently implemented than procedural sterilization and validation protocols [18]. Qualification-based differences were evident across multiple domains, with MDS practitioners demonstrating significantly better compliance in transmission knowledge, biofilm awareness and sterilization practices [19]. This suggests that postgraduate education contributes positively to infection control adherence; however, notable gaps persisted in both qualification groups. Structured continuing dental education programs, mandatory infection control audits and standardized monitoring systems may help bridge these gaps [20]. Overall, the findings highlight the need for strengthened infection control governance in private dental settings, particularly in peripheral regions. Policy-driven enforcement, periodic competency assessment and structured training modules are essential to ensure consistent adherence to evidence-based infection prevention standards.

## Conclusion:

Infection control practices among private dental practitioners in India demonstrate moderate compliance with significant deficiencies in aerosol management, sterilization validation, sharps handling and post-exposure protocols. Qualification level influences adherence with MDS practitioners showing better compliance; however, critical gaps persist across both groups. Thus, strengthening structured continuing education, routine monitoring and enforcement of standardized infection control guidelines is essential to enhance patient and occupational safety in private dental settings.
